# Antibiotic-degrading bacteria shape resistome dynamics and horizontal gene transfer potential in soils with contrasting properties

**DOI:** 10.1093/ismeco/ycaf246

**Published:** 2025-12-24

**Authors:** Zhi Mei, Chao He, Jose Luis Balcazar, Yuhao Fu, Qingyuan Dou, Yu Liu, Gerd Dercon, Xin Jiang, Martin Elsner, Fang Wang

**Affiliations:** State Key Laboratory of Soil & Sustainable Agriculture, Institute of Soil Science, Chinese Academy of Sciences, Nanjing 211135, P. R. China; Catalan Institute for Water Research (ICRACERCA), 17003 Girona, Spain; University of Chinese Academy of Sciences, Beijing 100049, P. R. China; State Key Laboratory of Soil & Sustainable Agriculture, Institute of Soil Science, Chinese Academy of Sciences, Nanjing 211135, P. R. China; Institute of Comprehensive Utilization of Resources, Kunming Metallurgical Research Institute Co., Ltd., Kunming 650021, P. R. China; Catalan Institute for Water Research (ICRACERCA), 17003 Girona, Spain; State Key Laboratory of Soil & Sustainable Agriculture, Institute of Soil Science, Chinese Academy of Sciences, Nanjing 211135, P. R. China; University of Chinese Academy of Sciences, Beijing 100049, P. R. China; State Key Laboratory of Soil & Sustainable Agriculture, Institute of Soil Science, Chinese Academy of Sciences, Nanjing 211135, P. R. China; University of Chinese Academy of Sciences, Beijing 100049, P. R. China; State Key Laboratory of Soil & Sustainable Agriculture, Institute of Soil Science, Chinese Academy of Sciences, Nanjing 211135, P. R. China; University of Chinese Academy of Sciences, Beijing 100049, P. R. China; University of Chinese Academy of Sciences, Beijing 100049, P. R. China; State Key Laboratory of Soil & Sustainable Agriculture, Institute of Soil Science, Chinese Academy of Sciences, Nanjing 211135, P. R. China; University of Chinese Academy of Sciences, Beijing 100049, P. R. China; Institute of Water Chemistry, Department of Chemistry, Technical University of Munich, Munich 85748, Germany; State Key Laboratory of Soil & Sustainable Agriculture, Institute of Soil Science, Chinese Academy of Sciences, Nanjing 211135, P. R. China; University of Chinese Academy of Sciences, Beijing 100049, P. R. China; Joint FAO/IAEA Centre of Nuclear Techniques in Food and Agriculture, International Atomic Energy Agency, Vienna 1400, Austria

**Keywords:** antibiotic-degrading bacteria, antimicrobial resistance genes, horizontal gene transfer, mobile genetic elements, DNA-SIP, soil microbiome, phenotype–genotype coupling

## Abstract

Soils act as both reservoirs and filters of antimicrobial resistance genes (ARGs); however, the ecological and genetic traits of antibiotic-degrading bacteria (ADB) and their interactions with nondegrading bacteria (NADB) across soil types remain poorly understood. In particular, the role of ADB in ARG dynamics and their potential contribution to horizontal gene transfer (HGT) are still underexplored. Here, we applied ^13^C-DNA stable isotope probing (DNA-SIP) combined with metagenomic sequencing to resolve active ADB from NADB in two contrasting soils: Ultisol and Mollisol. ADB harbored significantly more abundant and diverse chromosomal ARGs — especially multidrug and tetracycline resistance genes — often co-localized with mobile genetic elements (MGEs) and degradation genes, suggesting robust and regulated resistance strategies. In contrast, NADB relied more on plasmid-borne ARGs, reflecting flexible but potentially transient adaptation. Soil properties shaped both resistome composition and host taxa. Mollisol enriched enzymatic degraders such as *Lysobacter* and *Nocardioides*, while Ultisol favored stress-tolerant *Burkholderia*, which carried up to 34 ARGs and exhibited membrane-associated resistance. Notably, 89 ARGs or MGEs were found co-localized with degradation genes on assembled contigs, highlighting a strong potential for HGT. In addition, 24 high-potential ARG hosts were identified, including *Ralstonia pickettii* and *Saccharomonospora viridis*. These findings reveal that antibiotic degradation is embedded within complex, soil-specific resistome networks. This work enhances our understanding of ARG ecology and supports targeted mitigation strategies based on soil microbiome characteristics.

## Introduction

The dissemination of antimicrobial resistance genes (ARGs) has become a major concern for global public health and ecological stability [1–3]. The interaction between antibiotic residues and microbial activity plays a crucial role in shaping the physicochemical heterogeneity and biogeochemical processes that critically influence the distribution and spread of ARGs [4, 5]. Although tetracyclines, sulfonamides, and other antibiotics present in the environment can be degraded through microbial activity, thus reducing their concentrations in soil [6, 7], even low levels of these residues may exert enough selective pressure to drive the emergence and evolution of antimicrobial resistance [8]. Antibiotic-degrading bacteria (ADB), which play a primary role in the degradation of antibiotics, perform a dual ecological function. On the one hand, they reduce the selective stress exerted by antibiotics on microbial communities through biotransformation. On the other hand, they may act as reservoirs of ARGs and promote their dissemination through horizontal gene transfer (HGT) mediated by mobile genetic elements (MGEs) [9, 10]. HGT of ARGs can occur in virtually any environment where these genes are present. This process not only facilitates the broad dissemination of ARGs among diverse bacterial populations, but also enables the crossing of species barriers, promoting the transfer of resistance determinants from environmental, nonpathogenic bacteria to clinically relevant pathogens [11, 12]. Moreover, HGT is more likely to occur among phylogenetically related bacterial species, although environmental stressors, particularly the presence of antibiotics, can enhance the frequency of genetic exchange by promoting the mobilization and uptake of genetic material, thereby increasing the likelihood of ARG acquisition and stable integration into new bacterial hosts [13]. Therefore, under antibiotic selective pressure, the transfer of ARGs is particularly prevalent among human-associated bacteria [14], whereas the likelihood of resistance gene transfer from environmental bacteria, which inhabit distinct ecological niches and are phylogenetically distant from human pathogens, remains comparatively low. This suggests that once ARGs are acquired by pathogenic bacteria, the subsequent risk of dissemination among commensal and pathogenic species increases substantially [15].

In contrast, non-antibiotic-degrading bacteria (NADB), which coexist in the same environments but lack the metabolic capability to degrade antibiotics, exhibit substantial heterogeneity in their responses to antibiotic stress. While some microbial communities experience growth inhibition due to this metabolic limitation, others maintain ecological niche stability through intrinsic resistance mechanisms [16]. This functional differentiation suggests that ADB and NADB may play fundamentally distinct roles as hosts of ARGs and differ in their potential to facilitate ARG dissemination. However, accurately quantifying their relative contributions remains challenging, as traditional cultivation methods have historically captured only a small fraction of environmental microorganisms, giving rise to the long-standing “1% culturability paradigm” [17]. Although recent studies have shown that the proportion of culturable taxa can be substantially higher than previously assumed [18], a considerable fraction of microbial diversity remains uncultivated. DNA-stable isotope probing (DNA-SIP), therefore, offers a powerful culture-independent approach to identify active microbial functions in environmental samples. This method is based on the incorporation of isotopically labeled substrates into microbial DNA, thereby providing direct evidence of functional activity in uncultured microorganisms [19]. When combined with metagenomics, DNA-SIP enables the establishment of clear links between microbial identity and specific metabolic functions [20]. This approach has been successfully applied to trace complete metabolic pathways of organic pollutants, including sulfonamide antibiotics [21, 22]. However, studies employing DNA-SIP to reveal ARGs and their differences between ADB and NADB in soil remain limited.

Human pathogenic bacteria (HPB) act as critical vectors for the transmission of ARGs from environmental reservoirs to clinical settings, and their interactions with both ADB and NADB further exacerbate the risks associated with antimicrobial resistance. For instance, *Enterobacteriaceae* and *Staphylococcus* spp. can facilitate the dissemination of multidrug resistance genes (e.g. *bla*_CTX-M_ and *mcr-1*) across the environment-human interface [23]. Residual antibiotics in soil not only impose selective pressure that promotes HPB persistence, but may also enhance ARG enrichment within HPB populations through gene transfer networks mediated by ADB [11, 12]. However, the extent to which soil types influence the ecological differentiation of ADB functional communities and shape the spatial distribution patterns of ARG-HPB complexes remains poorly understood.

To address these limitations, this study employs sulfadiazine as a model antibiotic and applies DNA-SIP by introducing ^13^C-labeled sulfadiazine into different soil types to distinguish ADB from NADB. Functional ADB and NADB were successfully isolated from Ultisol and Mollisol, two representative soil types with contrasting physicochemical properties and a wide global distribution. Their distinct ecological characteristics offer a meaningful basis for exploring how soil type shapes the composition and resistance traits of antibiotic-degrading microbial communities. Subsequently, metagenomic sequencing was used to detect and compare the occurrence and distribution of ARGs, MGEs, and HPB within the isolated ADB and NADB communities. This study aims to explore: (i) the differences in ARG profiles of ADB across distinct soil types, (ii) whether variations in the abundance of MGEs and the efficiency of ARG transmission between ADB and NADB are regulated by soil-specific degradation functions, and (iii) the correlation between ARGs and HPB within ADB communities, as well as their potential biosafety risks. By addressing these aims, the study seeks to elucidate novel mechanisms through which soil–microbe functional interactions regulate the dissemination of ARGs, focusing on adaptive strategies such as the coupling of resistance genotypes with degradation phenotypes. The findings are expected to offer theoretical insights for the development of region-specific strategies for controlling antibiotic pollution and mitigating associated health risks.

## Materials and methods

### Soil preparation and antibiotic labeling treatments

The Ultisol (Ul) was collected from the Ecological Experimental Station of the Chinese Academy of Sciences in Yingtan, China, and the Mollisol (Mo) was sampled from farmland in Changchun, China. Both soils were air-dried and sieved through a 0.85 mm mesh. Their physicochemical properties are summarized in Table S2. To ensure experimental consistency, each soil type was thoroughly ground and homogenized before use, and four biological replicates were established for each soil type under identical incubation conditions. To activate indigenous microbial communities and stabilize soil conditions, both soils were pre-incubated for two weeks at 25°C in the dark, with moisture adjusted every two days. After this pre-incubation, sulfadiazine was added to the soils at a final concentration of 20 mg/kg to initiate the labeling experiment. Two types of SDZ were used: unlabeled ^12^C-sulfadiazine and ^13^C-labeled sulfadiazine (with all six carbon atoms of the benzene ring labeled; Cambridge Isotope Laboratories, USA). Each soil type (Ul and Mo) was thus divided into two treatments: one added with ^12^C-SDZ and the other with ^13^C-SDZ. A stock solution of each antibiotic (200 mg/L in methanol) was used for spiking. After spiking, the soils were further incubated under the same conditions as the pre-incubation. The goal of the ^13^C treatment was to trace the incorporation of labeled carbon into the DNA of sulfadiazine-degrading bacteria using DNA-SIP.

### DNA extraction and density gradient ultracentrifugation

Day 14 was selected for DNA extraction, as this time point corresponded to 84% - 90% sulfadiazine degradation in soil, according to previous studies conducted by our team (Fig. S1). Moreover, based on previous experiments, the overall microbial diversity (Shannon index) in both Mollisol and Ultisol soils remained relatively stable from Day 1 to Day 60 under sulfadiazine treatment (Fig. S2). Therefore, soil sampling for the current study was conducted at Day 14, representing the mid-incubation period. This indicates that most of the ^13^C-labeled antibiotic has likely been metabolized, allowing sufficient incorporation of ^13^C into the DNA of active degraders — crucial for successful DNA-SIP analysis. DNA was isolated from these samples using the FastDNA Spin Kit, following the manufacturer’s protocol (MP Biomedicals, CA, USA). The concentration of extracted DNA was determined using a Qubit® 3.0 Fluorometer (Thermo Fisher Scientific Inc., USA), and aliquots were stored at −80°C for downstream processing. To separate ^13^C-labeled DNA, the extracted genomic material was subjected to cesium chloride (CsCl) density gradient ultracentrifugation, following the protocol described in previous research [19, 24]. Briefly, 3000 ng of DNA was diluted in 100 μL gradient buffer (GB), then mixed with 4.9 ml CsCl solution (density: 1.85 g/ml) and 0.9 ml GB in a 15 ml ultracentrifuge tube. This mixture resulted in a final buoyant density of 1.725 g/ml. The tubes were ultracentrifuged at 45 000 rpm (≈190 000 g) for 45 h at 20°C using a Beckman VTi 65.2 vertical rotor. These conditions are routinely used in DNA-SIP experiments with CsCl density gradients and have been well established and validated in our laboratory and other studies [24]. Following centrifugation, 15 gradient fractions (300 μL each) were carefully collected from the tube base. The buoyant density of each fraction was measured using a handheld digital refractometer, and DNA was precipitated using PEG 6000 and 70% ethanol. After purification, DNA was resuspended in 30 μL sterile TE buffer. Fractions enriched in ^13^C-labeled DNA — presumably derived from sulfadiazine-degrading microorganisms — were identified by quantifying the abundance of 16S rRNA gene copies across the gradient [25]. Fractions showing distinct peaks in gene abundance within higher-density zones were considered indicative of successful ^13^C labeling. Accordingly, these heavy fractions were defined as originating from ADB, whereas the corresponding light ^12^C-DNA fractions, representing microorganisms that did not incorporate ^13^C under the tested conditions, implying that they were either not engaged in sulfadiazine catabolism or exhibited negligible metabolic activity, were operationally defined as NADB. All procedures were conducted in triplicate to ensure data robustness and reproducibility.

### Metagenomic sequencing and data analysis

Heavy-layer DNA fractions extracted from soils treated with ^12^C-sulfadiazine and ^13^C-sulfadiazine were subjected to metagenomic sequencing performed by Majorbio Biomedical Technologies Ltd. DNA fragments of ~400 base pairs (bp) were generated and used to construct paired-end libraries using the NEXTFLEX Rapid DNA-Seq kit (Bioo Scientific, Austin, TX, USA). Sequencing was carried out on an Illumina NovaSeq platform (Illumina Inc., San Diego, CA, USA). Due to soil type — dependent variation in microbial responses to antibiotic exposure, unequal quantities of ^13^C- and ^12^C-labeled DNA were sometimes recovered despite equal total DNA input. To meet the minimum requirement for library construction in cases of low DNA yield, multiple parallel fractions were pooled prior to sequencing. Clean reads were processed by quality filtering, including demultiplexing, removal of low-quality bases, and exclusion of host-derived sequences. Subsequently, reads were assembled into contigs using MEGAHIT (v1.1.2), and only contigs longer than 500 bp were retained. Open reading frames (ORFs) were predicted using Prodigal, and those annotated as ARGs using the DeepARG-LS model (e-value ≤ 1e^−10^; minimum identity 50%) were termed ARG-like ORFs [26]. These ARG-like ORFs were clustered into antibiotic resistance contigs (ARCs) [27, 28]. Sequencing depth per sample ranged from 78 to 121 million reads, providing a theoretical detection threshold in relative abundance between 1.28 × 10^−8^ and 8.26 × 10^−9^, below which taxa may not be reliably detected.

The abundance of ARG-like ORFs was quantified as coverage (×/Gb). Clean reads were mapped to the ARG-like ORFs using BWA (v0.7.17, bwa-mem algorithm) [29] (https://bio-bwa.sourceforge.net/), and mapped read counts per sample were recorded. Abundance within each ARG subtype or type was calculated following the formula below [30]:


(1) \begin{align*} &\mathrm{Abundance}\left(\mathrm{coverage},\times /\mathrm{Gb}\right)\\&\qquad=\sum_1^{\mathrm{n}}\frac{{\mathrm{N}}_{\mathrm{mapped}\ \mathrm{reads}}\times{\mathrm{L}}_{\mathrm{reads}}/{\mathrm{L}}_{\mathrm{ARG}-\mathrm{like}\ \mathrm{ORF}\ \left(\mathrm{ARC}\right)}}{\mathrm{S}} \end{align*}

${N}_{mapped\ reads}$ , the number of reads aligned to ARG-like ORFs; ${L}_{reads}$, the read length set at 150 bp; ${L}_{ARG- like\ ORF\ (ARC)}$, the length (bp) of the specific ARG-like ORF sequence; *n*, the total count of ARG-like ORFs assigned to the same ARG type, and *S* indicates the overall size of the metagenomic dataset.

### Mobility and taxonomic annotation of antibiotic resistance contigs

To assess the potential mobility of ARGs, ARCs were analyzed to determine their genetic location (plasmid vs. chromosome) using PlasFlow (v1.1) with a threshold of 0.7. Subsequently, the amino acid sequences of all ORFs identified within ARCs were aligned against the MGEs90 database using DIAMOND (v2.0.15.153) (e-value ≤ 1e^−5^, identity ≥ 25%, coverage ≥ 40%) [31], identifying MGE-like ORFs. Their abundance was calculated following the same approach as for ARGs, using equation (1).

For taxonomic annotation, ORFs from ARCs were aligned to the NCBI NR database (version nr_20200306) using DIAMOND (v2.0.15.153) with an e-value cutoff of 1e^−5^ [32]. A voting strategy was employed: if over 50% of the ARG-like ORFs on an ARC were annotated to the same taxonomic unit, the ARC was assigned to that taxon at the appropriate domain/kingdom/phylum/class/order/family/genus level. Species-level annotations were further compared against the HPB database to identify and quantify potential pathogens [31, 33].

### Statistical analysis

For data analysis, R (version 4.2.3) was utilized for initial processing. Heatmaps were generated using packages such as “plyr,” “ggplot2,” and “stringr” to visualize patterns of ARGs, community composition, and relative abundances across treatments. Histograms and line graphs were created with GraphPad Prism 9. Correlation matrices visualized in Gephi (version 0.10.1) illustrated pairwise relationships. Linear discriminant analysis Effect Size (LEfSe) identified significantly enriched ARG hosts between groups (LDA score > 2.0, *P* < 0.05), with a maximum of eight groups analyzed for statistical robustness using Python (version 3.9.13). Principal coordinates analysis (PCoA) based on Bray-Curtis dissimilarities was used to visualize differences in ARG subtype abundances among samples, and statistical significance was assessed using Adonis tests. Group differences in ARG abundances were evaluated with the Kruskal-Wallis test (*P <* 0.05). Procrustes analysis, using PCA for ordination, assessed correlations between changes in ARG abundances and host composition.

## Results

### Relative abundance distribution of 16S rRNA gene in different buoyancy density layers in Ultisol and Mollisol

**Figure 1:**
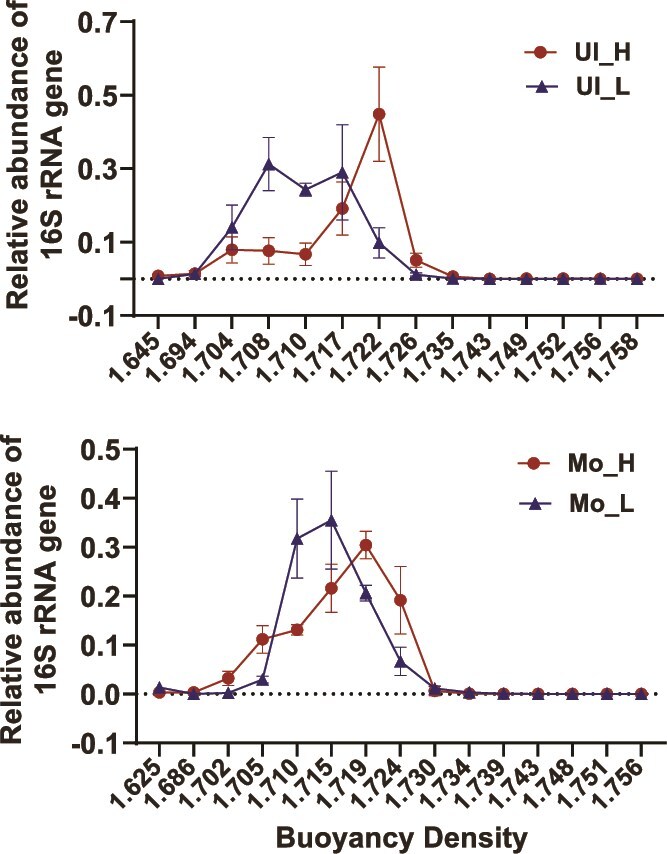
Relative abundance of 16S rRNA genes in ^13^C-labeled (H, heavy DNA) and ^12^C-labeled (L, light DNA) fractions across different buoyant density layers in Ultisol (Ul) and Mollisol (Mo) soils.

The distribution of 16S rRNA genes in ^13^C-labeled DNA (^13^C-DNA) and unlabeled DNA across different buoyant density fractions is shown for both Ultisol and Mollisol (Fig. 1). The red curve (^13^C-DNA) exhibits clear enrichment in higher buoyant density fractions compared to the blue curve (unlabeled DNA), consistently observed in both soil types. This distinct separation confirms the successful isolation of ^13^C-labeled DNA from control DNA under ultracentrifugation. The shift in buoyant density fractions further demonstrates that ^13^C-labeling effectively distinguishes microbial DNA assimilating ^13^C substrates in Ultisol and Mollisol. This separation enables the subsequent analysis of active ^13^C-assimilating microbes in both soil types.

### Abundance and distribution of ARGs carried by ADB and NADB

Metagenomic sequencing of Ultisol and Mollisol samples revealed distinct assembly characteristics. In Ultisol, contigs longer than 300 bp ranged from 594 690 to 936 774, with the largest contig reaching 269 760 bp. Predicted open reading frames (ORFs >102 bp) ranged from 894 796 to 1 331 999. In contrast, Mollisol showed greater assembly depth, with 700 100 – 1 627 740 contigs and up to 2 104 330 ORFs (Tables S3 and S4).

**Figure 2:**
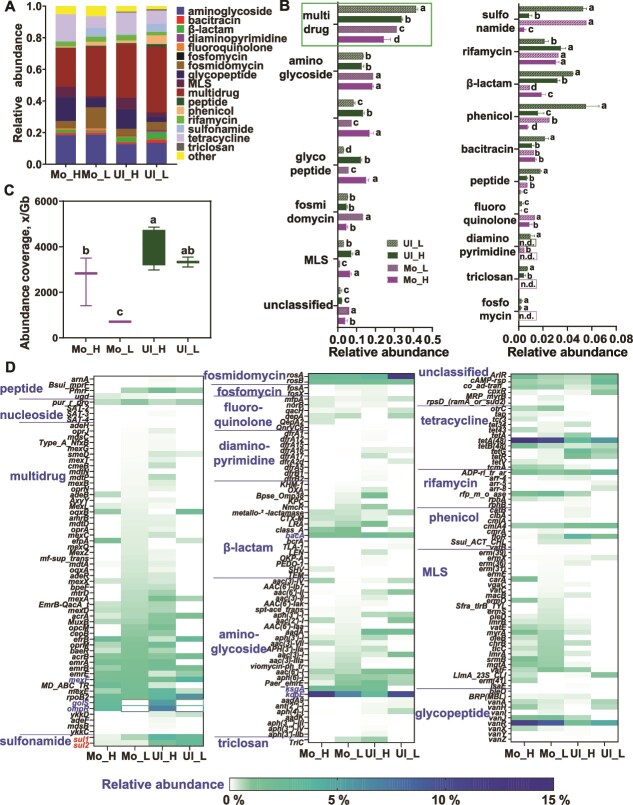
Antimicrobial resistance genes (ARGs) in different soils. (A) Distribution of ARGs across antibiotic resistance classes. (B) Differences in the relative abundance of ARGs among soil types and DNA fractions. (C) Total abundance of ARGs in different soils. (D) Relative abundance of ARG subtypes in different soils. Ul, Ultisol; Mo, Mollisol; H, heavy DNA fraction (DNA of sulfadiazine-degrading microorganisms); L, light DNA fraction (DNA of non-sulfadiazine-degrading microorganisms). Different lowercase letters indicate significant differences among treatments (*P <* 0.05). “n.d.” indicates parameters with no detectable signals.

A total of 19 ARG types and 198 ARG subtypes were detected in both soil types. Among these, 17 ARG types differed significantly in relative abundance between Ultisol and Mollisol (Fig. 2A and B). Overall, Ultisol exhibited significantly higher total ARG abundance in ADB than Mollisol (Fig. 2C, *P <* 0.05). ADB communities harbored more abundant ARGs than NADB in both soils. Multi-drug resistance (MDR) genes were the most dominant, accounting for 21% – 25% in Mollisol and 27% – 34% in Ultisol (Fig. 2B). Interestingly, the relative abundances of MDR, sulfonamide, and phenicol resistance genes were lower in ADB than in NADB. In contrast, tetracycline, glycopeptide, and macrolide-lincosamide-streptogramin (MLS) resistance genes were enriched in ADB. Aminoglycoside ARGs did not differ significantly between ADB and NADB, although they were more abundant in Mollisol overall. Notably, β-lactam resistance genes exhibited a soil-specific trend: higher in ADB than NADB in Mollisol, but the reverse in Ultisol.

**Figure 3:**
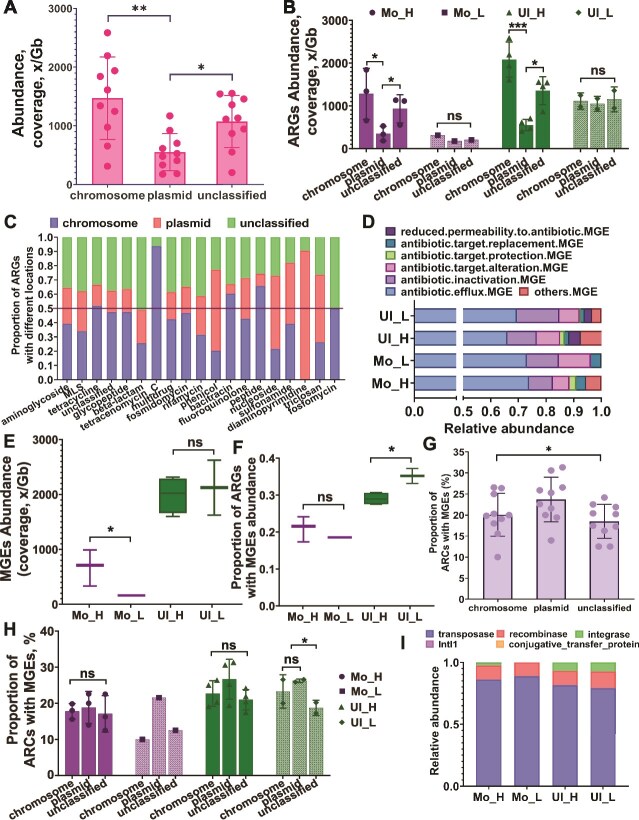
Distribution of antimicrobial resistance genes (ARGs) and mobile genetic elements (MGEs) in different genomic contexts and soil types. (A) Total abundance of ARGs in chromosomal, plasmid, and unclassified DNA. (B) Abundance of ARGs in these genomic categories across different soil types. (C) Relative abundance of ARG classes in chromosomal, plasmid, and unclassified DNA. (D) Relative abundance and proportion of ARGs co-occurring with various MGE mechanisms. (E) Absolute abundance of MGEs in different soil samples. (F) Relative abundance of MGEs in different soil samples. (G and H) Ratio of MGEs in chromosomal, plasmid, and unclassified DNA. (I) Relative abundance of different types of MGEs. Asterisks indicate statistical significance: ^*^P < 0.05, ^**^P < 0.01, ^***^P < 0.001.

Despite a lower overall abundance of several ARG types, ADB communities in the Mollisol soil exhibited a significantly higher ARG subtype richness than NADB communities (*P <* 0.05), as shown in Fig. S3. The most prevalent ARGs included *tetA(48)*, *vanR*, and *kdpE*, with cumulative coverages of 361 Gb, 317 Gb, and 323 Gb, respectively, across Ultisol and Mollisol (Fig. 2D). Chromosomal ARGs were significantly more abundant than plasmid-borne ones, especially in ADB (Fig. 3A and B, *P*  *<* 0.05). No such difference was observed in NADB. ARG types such as bacitracin, nucleoside, peptide, diaminopyrimidine, and triclosan were primarily plasmid-associated, whereas others were mainly chromosomal (Fig. 3C).

The total abundance of MGEs was significantly higher in Ultisol than in Mollisol (Fig. 3E). In Ultisol, MGEs were more abundant in NADB than ADB; the opposite trend was seen in Mollisol, though differences were not statistically significant (Fig. 3F). Most MGEs were transposons (Fig. 3I), and plasmid-borne MGEs were more prevalent than chromosomal ones in Ultisol (Fig. 3G and H). MGEs involved in antibiotic efflux were the most common, followed by those associated with target alteration — the latter being more abundant in NADB. MGEs related to reduced permeability were exclusively found in Ultisol, with a higher proportion in ADB (Fig. 3D).

### Divergent ARG profiles and dominant taxa in ADB and NADB

**Figure 4:**
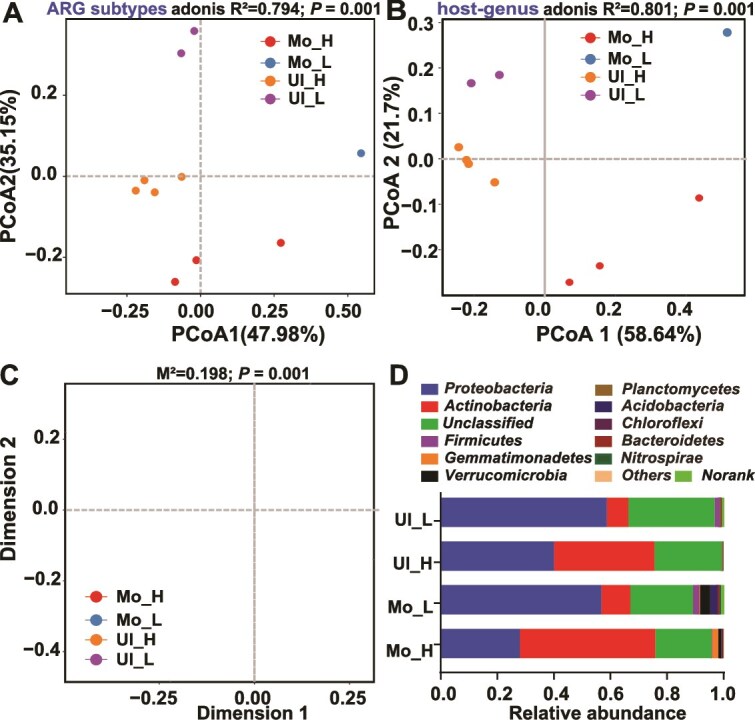
Correlation between antimicrobial resistance genes (ARGs) and microbial community structure. (A and B) principal coordinates analysis (PCoA) of ARG subtypes (A) and ARG-host associations (B) based on Bray–Curtis dissimilarity. (C) Procrustes analysis showing the concordance between antibiotic-degrading vs. nondegrading bacteria and the antibiotic resistome, based on NMDS of ARG and microbial relative abundances (M^2^: Procrustes sum of squares, permutations = 999). (D) Relative abundance of ARG-hosting microorganisms at the phylum level.

ADB and NADB microbial communities were not entirely distinct but shared overlapping taxa (Fig. S4). Nonetheless, both antibiotic degradation activity and soil type were key factors contributing to differences in microbial community structure and ARG distribution (Fig. 4A and B). In Ultisol, community shifts associated with antibiotic metabolism were primarily observed along the PCoA2 axis, whereas in Mollisol, changes were evident along both PCoA1 and PCoA2. Procrustes analysis further confirmed a strong correlation between ARG profiles and ARG-hosting microbial communities (*P* = 0.001; Fig. 4C), underscoring the linkage between functional and taxonomic shifts during SDZ degradation.

*Proteobacteria* and *Actinobacteria* were the dominant phyla in both ADB and NADB. While *Actinobacteria* were enriched in ADB, *Proteobacteria* were more abundant in NADB (Fig. 4C). In Ultisol, *Firmicutes* were not detected among ARG-hosting ADB, while in Mollisol they accounted for ~ 0.1% of the ADB. By contrast, *Firmicutes* constituted ~ 2% of the NADB in both soils. *Gemmatimonadetes* and *Verrucomicrobia* appeared only in Mollisol NADB, with *Gemmatimonadetes* more abundant in ADB (2.2%) than in NADB (0.3%). Dominant ADB genera included *Actinomadura* and *Microbispora* (up to 10% and 3%), and *Streptomyces* (4.5% – 4.7%), none of which were found in NADB. Massilia was present in both groups, with slightly higher abundance in NADB. Co-occurrence analysis revealed these genera carried multiple ARGs (*Actinomadura*: 29, *Streptomyces*: 27, *Microbispora*: 23, *Massilia*: 21), while *Burkholderia*, detected only in Ultisol (4.4% – 6.0%), harbored the most ARGs (34). ARG and ARB diversity was higher in NADB than ADB in Ultisol, but the opposite was observed in Mollisol.

**Figure 5-1:**
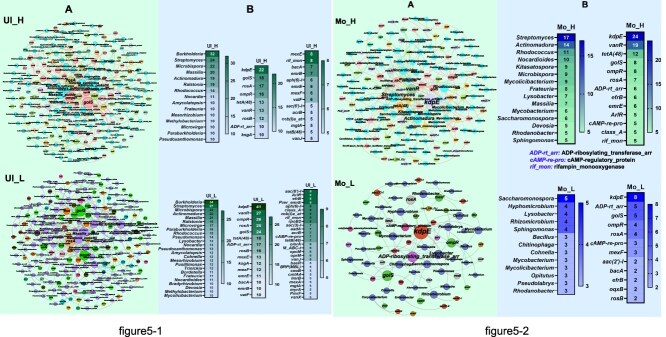
Co-occurrence network of antimicrobial resistance genes (ARGs) and ARG-hosting bacteria in Ultisol. (A) Network analysis showing co-occurrence patterns between antibiotic resistance genes (ARGs), mobile genetic elements (MGEs), and bacterial genera in Ultisol. Edges represent strong (Spearman’s ρ > 0.7) and significant (*P <* 0.05, n > 5) correlations. Only bacteria associated with ARGs or MGEs are shown; intra-bacterial correlations are excluded. (B) Number of connections (degree) for each ARG subtype or ARG-hosting bacterial genus in the network. Ul_H: DNA of sulfadiazine-degrading bacteria; Ul_L: DNA of non-sulfadiazine-degrading bacteria. **Figure 5-2**. Co-occurrence network of antimicrobial resistance genes (ARGs) and ARG-hosting bacteria in Mollisol. (A) Network analysis showing co-occurrence patterns between ARGs, MGEs, and bacterial genera in Mollisol. Edges represent strong (Spearman’s ρ > 0.7) and significant (*P <* 0.05, n > 5) correlations. Only bacteria associated with ARGs or MGEs are shown; intra-bacterial correlations are excluded. (B) Number of connections (degree) for each ARG subtype or ARG-hosting bacterial genus in the network. Mo_H: DNA of sulfadiazine-degrading bacteria; Mo_L: DNA of non-sulfadiazine-degrading bacteria.

ARG richness and distribution varied markedly across soils and bacterial groups. In Ultisol, NADB exhibited greater ARG diversity than ADB, with 165 versus 133 ARGs detected, and ARGs spread across 148 versus 76 genera, respectively (Fig. 5-1A). In contrast, Mollisol ADB showed higher diversity, with 75 ARGs in 81 genera compared to only 38 ARGs in 36 NADB genera. Widely distributed ARGs included *kdpE, vanR*, and *tetA(48)*, with *kdpE* present in 22 – 41 genera across groups. In Ultisol, ARGs such as *golS*, *rosA*, *ompA*, *vanR*, and *tetA(48)* were more prevalent in NADB than in ADB (Fig. 5-1B). In Ultisol, nine ARG subtypes were carried by more than 10 ADB genera, while more than 10 NADB genera harbored 15 ARG subtypes, and 15 ADB genera carried more than 10 ARGs, whereas 25 NADB genera harbored more than 10 ARGs. Bacterial genera carrying more than 20 ARGs in Ultisol included *Burkholderia*, *Streptomyces*, *Microbispora, Actinomadura*, and *Massilia* (Fig. 5-1B). These genera were present in both ADB and NADB communities (Fig. 5-1A). In Mollisol, ADB exhibited greater ARG diversity and abundance compared to NADB. Among the ADB genera, *Streptomyces*, *Actinomadura*, *Rhodococcus*, and *Nocardioides* carried more than 10 ARGs. In contrast, *Saccharomonospora* was the NADB genus with the most ARGs (5), and was the only one harboring both *vanR* and *tetA(48*), though these two ARGs were also detected in 18 and 11 other NADB genera, respectively. The most widespread ARG in Mollisol was *kdpE*, found in 24 NADB and eight ADB genera (Fig. 5-2B).

**Figure 6:**
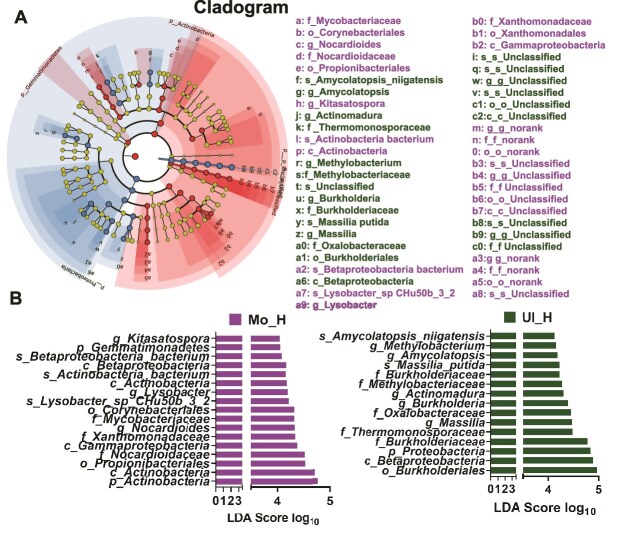
Discriminant analysis of ARG-hosting microorganisms. (A) LEfSe-generated phylogenetic cladogram. Each colored branch represents a distinct group, with red nodes indicating ARG-hosting taxa that significantly contribute to differences in the red group, and yellow nodes representing taxa with no significant group contribution. (B) LDA bar plot of ARG-hosts with significant differential abundance between groups. Bar length corresponds to the LDA score, indicating the magnitude of the contribution to group separation.

LEfSe analysis identified distinct microbial signatures between ADB communities across soil types. In Mollisol, taxa enriched in ADB included *Lysobacter*, *Kitasatospora*, and *Nocardioides*, as well as *Lysobacter* sp*. CHu50b_3_2* and *Actinobacteria* bacterium (Fig. 6A). In Ultisol, ADB was characterized by *Burkholderia*, *Actinomadura*, *Amycolatopsis*, *Massilia*, and *Methylobacterium*, with representative species including *Amycolatopsis niigatensis* and *Massilia putida* (Fig. 6B). These indicator taxa likely contribute to soil-specific differences in ARG dissemination and degradation potential.

### Co-localization of ARGs with MGEs and plasmid signatures

To further investigate the potential for HGT of ARGs in Ultisol and Mollisol, we examined the co-localization of ARGs with MGEs and plasmid signatures in assembled contigs (Tables 1 and S1). A total of 89 contigs carrying ARGs (e.g. *kdpE*, *rpoB2*, and *NmcR*) were annotated with plasmid origin and/or associated MGEs, including transposases, integrases, and recombinases. Among them, 30 contigs were predicted to originate from plasmids, 19 from chromosomes, and 40 were of unclassified origin. The most frequently co-localized MGEs were transposases (n = 56), followed by recombinases (n = 21) and integrases (n = 7). Multiple contigs (e.g. k97–1 443 424, k97–151 772) were annotated as plasmid-derived and carried MDR genes or β-lactamases, suggesting a high potential for horizontal dissemination. Additionally, contigs of unclassified origin (e.g. k97–516 524, k97–283 951) also harbored ARGs and were linked with MGEs such as *IS1380* family transposases and ssDNA recombinases, indicating possible cryptic mobility pathways.

**Table 1:** Number of contigs harboring ARG and MGE types in plasmid-, chromosome-, and unclassified-predicted categories.

MGE/ARG type	Number ofantibiotic resistance contigs		
	Plasmid	Chromosome	Unclassified
ARG-MDR	1	-	2
ARG-rifampin	1	-	-
ARG-β-lactam	-	-	1
MGE-transposase	23	7	26
MGE-integrase	2	3	2
MGE-recombinase	3	9	9

### Distribution and ARG profiles of HPB in ADB and NADB in Ultisol and Mollisol

**Figure 7:**
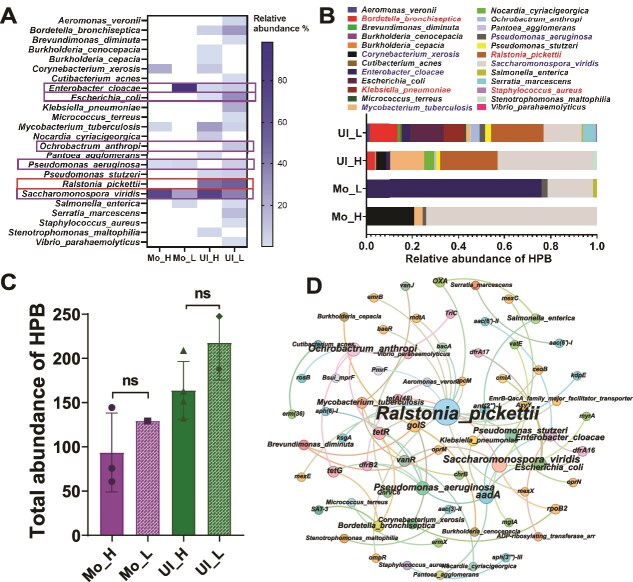
Distribution and associations of human pathogenic bacteria (HPB) carrying ARGs. (A and B) The relative abundance distribution of HPB among antibiotic-resistant bacteria in different soil samples. (C) The total absolute abundance of HPB among antibiotic-resistant bacteria in different soil samples. (D) Network analysis showing co-occurrence patterns between ARGs, MGEs, and ARG-hosting HPB. Edges represent strong (Spearman’s ρ > 0.7) and significant (*P <* 0.05, n > 5) correlations. Only HPB taxa associated with ARGs or MGEs are shown; intra-HPB correlations are excluded.

A total of 24 HPB were detected across both soil types (Fig. 7A). While the relative abundance of HPB was consistently higher in NADB than in ADB in both soils, the differences were not statistically significant (Fig. 7C). In Ultisol, 14 HPB were found in ADB and 19 in NADB (Fig. 7A). *Saccharomonospora viridis* and *Ralstonia pickettii* were the most abundant HPB in both bacterial groups. Notably, the relative abundance of *S. viridis* in ADB was 2.5 times higher than in NADB in Ultisol, and 3.7 times higher in Mollisol (Fig. 7B). In Mollisol, only four HPB were shared between ADB and NADB. *Corynebacterium xerosis* and *Mycobacterium tuberculosis* were exclusively detected in ADB, whereas *Enterobacter cloacae* was unique to NADB (Fig. 7A). *R. pickettii*, detected only in Ultisol, harbored the highest number of ARGs (16), including 11 multidrug resistance genes, as well as genes conferring resistance to aminoglycosides (2), bacitracin (1), β-lactams (1), and peptides (1). It was more abundant in NADB than in ADB (Fig. 7D).

Among HPB, the most widely distributed ARG was *aadA*, found in six species: *E. cloacae*, *Escherichia coli*, *Klebsiella pneumoniae*, *Pantoea agglomerans*, *Pseudomonas aeruginosa*, and *Pseudomonas stutzeri*. Of these, only *P. aeruginosa* was present in Mollisol. The second most widespread gene, *golS*, was detected in several Ultisol-derived HPB, including *Brevundimonas diminuta*, *Burkholderia cenocepacia*, *Burkholderia cepacia*, and *R. pickettii*, but was absent in Mollisol. For tetracycline resistance, *tetR* and *tetG* were identified in four and three pathogenic species, respectively, including *Bordetella bronchiseptica*, *Ochrobactrum anthropi*, *P. aeruginosa*, and *P. stutzeri*. Except for *P. aeruginosa*, none of these species were detected in Mollisol (Fig. 7A).

## Discussion

The ecological differentiation of ADB is strongly governed by soil type, which shapes microbial community structure and, in turn, influences the spatial distribution of ARGs. While ADB and NAD exhibit distinct functional traits, several core genera — including *Massilia*, *Actinomadura*, and *Burkholderia* — were consistently detected in both groups. These taxa exhibit broad metabolic versatility: *Massilia* is recognized as a keystone species in various soil ecosystems [34]; *Actinomadura* is involved in a range of enzymatic processes [35]; and *Burkholderia* contributes significantly to nitrogen cycling [36]. Their persistence across both degrading and nondegrading communities suggests that antibiotic degradation may be embedded within a broader metabolic repertoire, enabling functional maintenance even in environments lacking direct antibiotic pressure. Since the DNA-SIP experiment employed only ^13^C-labeled sulfadiazine as a model antibiotic, the identified ADB should be interpreted as active degraders of sulfadiazine under the tested conditions rather than as universal antibiotic degraders. Nevertheless, many of these taxa exhibit well-documented capabilities to metabolize diverse xenobiotic compounds, suggesting that their functional potential may extend beyond sulfadiazine degradation under varying environmental conditions.

### Environmental factors of antibiotic resistance contig and microbial distribution across soil types

Soil physicochemical properties further modulate ARG distribution. In Ultisol, characterized by high acidity (pH 5.4) and low organic matter (0.47%), microbial diversity is limited; however, these challenging conditions selectively favor ADB enrichment. PCoA showed that ARGs and their hosts in Ultisol clustered mainly along the PCoA2 axis (Fig. 4B), suggesting that antibiotic gradients and other environmental pressures shape microbial assembly within specific ecological dimensions. In contrast, Mollisol, with its higher organic matter (2.2%), supports a more diverse and complex microbial network [37, 38]. Previous studies have shown that soil pH is negatively correlated with overall ARG abundance, likely because inner-membrane-associated ARGs tend to be enriched in acidic soils, whereas higher pH levels favor greater overall microbial diversity [39, 40]. These findings, together with differences in organic matter content and soil pH, suggest that both nutrient availability and acidity act as key environmental drivers shaping the distinct ARG and microbial patterns observed between the two soil types. Here, ARG distributions reflect the influence of multiple environmental axes, where co-occurring factors buffer individual stressors such as antibiotic presence, promoting ARG persistence through co-evolution with microbial hosts [41].

### Functional and taxonomic divergence of degrading communities in Ultisol and Mollisol

Soil type also shaped the composition and enrichment of specific degrading taxa. The relative abundances of *Lysobacte*r, *Kitasatospora*, and *Nocardioides* were higher in Mollisol compared to Ultisol (Fig. S4). *Lysobacter*, known for secreting antimicrobial peptides [42], contributes to β-lactam degradation and has been positively associated with higher pH and clay content [43, 44]. *Nocardioides* has shown potential for HGT, which may contribute to ARG dissemination [45, 46]. In Ultisol, however, the enrichment of *Actinomadura*, *Massilia*, and *Burkholderia* points to adaptation to acidic conditions (Fig. S4). *Burkholderia* not only harbors the highest number (34 total; Fig. 5-1B) of ARGs, but also rapidly develops resistance phenotypes under tetracycline stress through multidrug efflux pump systems [47, 48]. This remarkable adaptability in Ultisol (relative abundance: 4.4%–6.0%) contributes significantly to the dissemination of ARGs.

Notably, *Streptomyces* — a genus traditionally associated with antibiotic production — was exclusively detected in the ADB fraction, suggesting potential involvement in ARG exchange via interspecies genetic transfer [49, 50]. Furthermore, the absence of *Firmicutes* in Ultisol (detection limit <0.1%) is consistent with their known sensitivity to low pH [51], while the presence of *Verrucomicrobia* in Mollisol (abundance: 1.2% – 1.8%) likely reflects their preference for environments rich in organic matter. These compositional patterns, together with metagenomic evidence — including the annotation of antibiotic-degradation genes, co-occurrence analysis of ARGs and MGEs, and host-ARG co-localization — suggest that Mollisol communities are more reliant on enzymatic degradation pathways, while Ultisol communities favor membrane-associated resistance mechanisms such as efflux pumps.

### Mobilization pathways of ARGs differ between ADB and NADB across soil types

The differing genomic localization of ARGs between ADB and NADB communities reflects distinct ecological resistance strategies. In ADB, ARGs (e.g. *kdpE*, *NmcR*, and *rpoB2*) were frequently chromosomally encoded and co-localized with degradation-related genes (e.g. *ata*, *sadA*, and *emaA*) and MGEs on the same contigs, suggesting a coordinated and stable integration of resistance and catabolic functions into the bacterial core genome. This pattern is consistent with established knowledge that efflux pumps — the predominant resistance mechanism — are typically chromosomal and comprise over 60% of chromosomally encoded resistance determinants [52, 53]. Notably, in Actinobacteria-dominated ADB communities, chromosomal ARGs were often located near secondary metabolite biosynthetic gene clusters, indicating an evolutionary coupling between antibiotic production and resistance [49].

By contrast, NADB communities relied more on plasmid-borne ARGs, especially those related to target protection and replacement, highlighting their dependence on mobile genetic platforms for rapid acquisition of broad-spectrum resistance via HGT [12, 54]. These differences suggest divergent ecological adaptations: ADB communities appear to favor stability and regulatory control via chromosomal integration, while NADB exploit plasmid-based plasticity for dynamic responses to environmental pressure.

MGEs further reinforce these strategies. In ADB-enriched environments, ARG dissemination is supported by the co-localization of ARGs with MGEs, including transposases, integrases, and recombinases — elements known to mediate gene recombination and mobilization under stress [55, 56]. The presence of MDR and β-lactamase genes on shared plasmid backbones (e.g. pOXA-48-like elements) or within integron cassettes facilitates co-selection and persistence under antibiotic pressure [57]. Furthermore, cryptic mobility pathways may exist, as suggested by ARGs on unclassified contigs harboring elements such as *IS1380* transposases and ssDNA recombinases.

Environmental context plays a key role: MGEs were more abundant in Ultisol than in Mollisol (Fig. 3E and F), likely due to harsher physicochemical stress — e.g. low pH and nutrient limitation — that intensifies selection for HGT-mediated adaptation [58, 59]. In NADB communities from Ultisol, elevated MGE levels reflect a stronger reliance on HGT, while in Mollisol, ADB may use MGEs primarily to disseminate degradation-related genes [60, 61]. This highlights the ecological specificity of ARG mobilization pathways. Finally, these patterns reflect a broader phenomenon where DNA-level functional potential often diverges from actual transcriptional activity. Similar discrepancies have been observed in complex microbial communities, including the human gut, where transcriptomic profiles exhibit greater inter-individual variation and stronger links to host phenotypes than genomic content [62, 63]. These results collectively support the view that gene co-localization on mobile elements is a central mechanism for ARG persistence and dissemination in ADB-dominated environments [57].

### Divergent ARG profiles and adaptive strategies between ADB and NADB communities

In addition to enhanced mobility, ADB and NADB communities exhibit distinct resistance strategies. Overall ARG abundance was significantly higher in ADB (*P <* 0.05), likely due to the dual advantage of antibiotic degradation and ARGs acquisition (Fig. S5). ADB taxa metabolize antibiotics while retaining ARGs that offer further protection. This pleiotropic function is also evident in some aminoglycoside degradation pathways. Specific ARGs such as *kdpE*, *golS*, and *rosA* were significantly enriched in ADB (Fig. 5-1), potentially due to their auxiliary roles in stress response systems such as ion regulation [64]. The co-occurrence of *vanR* and *tetA* with metabolically versatile Actinobacteria (e.g. *Streptomyces*, *Nocardioides*) also suggests a link between secondary metabolism and ARG retention. Soil type modulated ARG patterns: β-lactam resistance genes were more abundant in Mollisol than Ultisol (Fig. 2B, D), indicating that enzymatic degradation might be favored in organic-rich soils. In Mollisol, microbial communities may favor enzymatic degradation as a primary response to antibiotic exposure, while Ultisol communities may rely more on membrane-based resistance mechanisms, such as outer membrane protein modifications (e.g. *OmpA*) [64].

ADB and NADB also diverged in ARG types. NADB communities were enriched in broad-spectrum efflux genes (e.g. *oprJ*, *acrA-05*), providing generalized resistance in the absence of degradation capacity [65] (Fig. S6); In contrast, ADB relied more on specific enzymatic systems (e.g. β-lactamases), enabling direct detoxification of antibiotics. Gene-specific patterns further illustrate this divergence. *Sul1* and *floR* were significantly less abundant in ADB, suggesting that degradation may reduce the need for these genes. In contrast, *tetM*, *vanA*, and *ermB* were significantly enriched (*P <* 0.05), possibly due to their roles in detoxifying intermediates during degradation. Although *aac(6′)-Ib* abundance did not differ between groups, its levels were 43% higher in Mollisol (*P <* 0.05), potentially reflecting historical gentamicin application in regional agricultural practices.

### ADB community network connectivity and HPB abundance

ADB communities in Ultisol showed higher network connectivity than those in Mollisol. Moreover, the abundance of HPB was significantly greater in Ultisol. Although this may seem to contradict the notion that higher diversity enhances community stability and invasion resistance, high antibiotic pressure could be weaken network integrity, facilitating HPB colonization. This pattern likely reflects environmental filtering. The acidic, nutrient-poor conditions in Ultisol favor stress-tolerant HPB, such as *Ralstonia picketii*, which can survive at pH 4.5 – 5.5 and adapt to low-nutrient environments [66–68]. In contrast, the higher organic matter in Mollisol may support co-metabolic degradation of antibiotics, exemplified by *S. viridis*. Acid stress in Ultisol may also redirect microbial energy toward maintaining pH homeostasis rather than competition [69], while Fe(III) oxides can intensify extracellular electron competition and facilitate iron acquisition by HPB through siderophore hijacking [70]. Although HPB and degrading microbes in Ultisol may utilize overlapping carbon sources, low pH, nutrient limitation, and iron scarcity likely modulate their interactions. This supports the Stress Gradient Hypothesis, which posits that facilitative interactions become more prominent under increasing environmental stress [71].

In conclusion, this study elucidates how soil type shapes the ecological assembly and resistance strategies of ADB and NADB. Successful ^13^C-labeling enabled precise separation of ADB from NADB, revealing distinct metagenomic signatures between the two groups across Ultisol and Mollisol. ADB communities harbored more abundant and diverse chromosomal ARGs, particularly MDR and tetracycline resistance genes, often co-localized with MGEs and degradation genes, suggesting stable and regulated resistance strategies. In contrast, NADB showed greater reliance on plasmid-borne ARGs, indicating a more flexible but transient resistance potential. Soil type exerted a strong influence on ARG patterns and host taxa. Mollisol favored taxa like *Lysobacter* and *Nocardioides*, associated with enzymatic degradation, while Ultisol selected for stress-tolerant genera such as *Burkholderia*, which carried up to 34 ARGs and exhibited membrane-associated resistance mechanisms. High levels of co-localization between ARGs and MGEs (89 contigs) and the presence of high-potential ARG hosts (HPB) like *R. pickettii* and *S. viridis* underscore the risk of horizontal ARG dissemination. These findings highlight that antibiotic degradation is not an isolated function but embedded within complex, soil-dependent resistome dynamics, with implications for predicting ARG mobility and developing mitigation strategies. While these conclusions are robust within the scope of the experimental systems studied, they should be interpreted in light of the limited number of soil types tested. Nevertheless, the contrasting properties of Ultisol and Mollisol provide a valuable framework for understanding how environmental factors shape ARG dynamics, offering a solid foundation for future validation across broader ecological settings.

## Supplementary Material

Supplementary_Information_ycaf246

## Data Availability

The raw metagenomic sequencing data supporting the findings of this study have been deposited in the NCBI [GenBank] repository under the accession numbers PRJNA1274690 (http://www.ncbi.nlm.nih.gov/bioproject/PRJNA1274690).
