# Supplementary material for: Antibiotic-degrading bacteria shape resistome dynamics and horizontal gene transfer potential in soils with contrasting properties
Source: ISME Commun. 2025 Dec 24;6(1):ycaf246. doi: 10.1093/ismeco/ycaf246 (PMC12815267; doi:10.1093/ismeco/ycaf246)
Supplement: Supplementary_Information_ycaf246 [file supplementary_information_ycaf246.docx]

**Supplementary Information for:**

**“Antibiotic-Degrading Bacteria Shape Resistome Dynamics and Horizontal Gene Transfer Potential in Soils with Contrasting Properties”**

**Tables**

**Table S1** Contigs with K21449-associated antibiotic degradation genes (*ata, sada,* and *emaa*) detected with ARGs/MGEs and their corresponding contig identifiers.

| **Contig** | **Plasmid**  **predict** | **MGE/ARG**  **type** | **MGE/ARG_subtype** |
| --- | --- | --- | --- |
| k97-1443424 | plasmid | MDR | *kdpE* |
| k97-151772 | plasmid | rifampin | *rpoB2* |
| k97-516524 | unclassified | β-lactam | *NmcR* |
| k97-662749 | unclassified | MDR | *kdpE* |
| k97-283951 | unclassified | MDR | *kdpE* |
| k97-811168 | chromosome | transposase | MULTISPECIES:_transposase_[Enterobacteriaceae] |
| k97-102744 | chromosome | transposase | IS110_family_transposase_[Pedosphaera_parvula] |
| k97-598026 | chromosome | transposase | IS110_family_transposase_[Fimbriiglobus_ruber] |
| k97-202423 | chromosome | integrase | integrase_[Geothermobacter_sp._HR-1] |
| k97-1322461 | chromosome | recombinase | recombinase_family_protein_[Arthrobacter_sp._MWB30] |
| k97-1361516 | chromosome | transposase | transposase_[Alloactinosynnema_sp._L-07] |
| k97-1274833 | chromosome | recombinase | tyrosine_recombinase_[Solirubrobacterales_bacterium_URHD0059] |
| k97-729571 | chromosome | recombinase | recombinase_RecB_[Helicobacter_cetorum] |
| k97-884554 | chromosome | transposase | IS1380_family_transposase_[Streptococcus_pneumoniae] |
| k97-1009945 | chromosome | recombinase | recombinase_RecA_[Rhodospirillales_bacterium_URHD0088] |
| k97-1060150 | chromosome | recombinase | DNA_recombinase_[Rhodococcus_rhodochrous] |
| k97-371431 | chromosome | transposase | Tn3_family_transposase_[Escherichia_coli] |
| k97-96624 | chromosome | integrase | phage-related_integrase_[Pandoraea_sp._SD6-2] |
| k97-248302 | chromosome | transposase | IS630_family_transposase_[Bradyrhizobium_ottawaense] |
| k97-42317 | chromosome | recombinase | tyrosine_recombinase_XerD_[Bdellovibrio_sp._SKB1291214] |
| k97-671109 | chromosome | integrase | site-specific_integrase_[Zavarzinella_formosa] |
| k97-1165780 | chromosome | recombinase | recombinase_family_protein_[Nakamurella_panacisegetis] |
| k97-798076 | chromosome | recombinase | recombinase_family_protein_[Phaeospirillum_fulvum] |
| k97-146615 | chromosome | recombinase | recombinase_RecA_[Azoarcus_sp._SY39] |
| k97-1157359 | plasmid | integrase | site-specific_integrase_[Acidobacteriaceae_bacterium_URHE0068] |
| k97-448638 | plasmid | recombinase | recombinase_family_protein_[Streptomyces_sp._HG99] |
| k97-1226970 | plasmid | transposase | transposase_[Meiothermus_chliarophilus] |
| k97-13878 | plasmid | transposase | IS605_family_transposase_OrfB_(plasmid)_[Bacillus_thuringiensis_serovar_chinensis_CT-43] |
| k97-720073 | plasmid | transposase | IS66_family_transposase_[Rhodopirellula_sallentina] |
| k97-1354805 | plasmid | recombinase | recombinase_RecB_[Helicobacter_jaachi] |
| k97-915875 | plasmid | transposase | IS3_family_transposase_[Xanthomonas_axonopodis] |
| k97-792062 | plasmid | transposase | IS481_family_transposase_[Bordetella_pertussis] |
| k97-280514 | plasmid | transposase | transposase_[Burkholderia_sp._D7] |
| k97-638684 | plasmid | transposase | ISKra4_family_transposase_[Burkholderia_sp._OK233] |
| k97-1442025 | plasmid | integrase | site-specific_integrase_[Methylibium_sp._Root1272] |
| k97-509410 | plasmid | transposase | transposase_[Brachybacterium_squillarum] |
| k97-1531842 | plasmid | transposase | IS110_family_transposase_[Acetobacter_pasteurianus] |
| k97-884346 | plasmid | transposase | IS1380_family_transposase_[Streptococcus_pneumoniae] |
| k97-73823 | plasmid | transposase | IS110_family_transposase_[Leptospira_borgpetersenii] |
| k97-1237728 | plasmid | recombinase | recombinase_RecQ_[Microbacterium_sp._RU33B] |
| k97-1108342 | plasmid | transposase | IS110_family_transposase_[Bradyrhizobium_yuanmingense] |
| k97-787932 | plasmid | transposase | transposase_[Magnetospirillum_gryphiswaldense] |
| k97-493751 | plasmid | transposase | MULTISPECIES:_transposase_[Halomonas] |
| k97-288011 | plasmid | transposase | transposase_[Methylomicrobium_album] |
| k97-501883 | plasmid | transposase | IS5/IS1182_family_transposase_[Streptomyces_pini] |
| k97-293903 | plasmid | transposase | MULTISPECIES:_transposase_[Halomonas] |
| k97-292967 | plasmid | transposase | transposase_[Turneriella_parva] |
| k97-251353 | plasmid | transposase | transposase_[Caldilinea_aerophila] |
| k97-539729 | plasmid | transposase | IS110_family_transposase_[Bradyrhizobium_neotropicale] |
| k97-619283 | plasmid | transposase | IS110_family_transposase_[Komagataeibacter_europaeus] |
| k97-702057 | plasmid | transposase | ISKra4_family_transposase_[Phormidesmis_priestleyi] |
| k97-704065 | plasmid | transposase | IS5/IS1182_family_transposase_[Antarctobacter_heliothermus] |
| k97-356762 | unclassified | transposase | IS630_family_transposase_[Pseudonocardia_sp._EC080619-01] |
| k97-711061 | unclassified | transposase | IS1380_family_transposase_[Streptococcus_pneumoniae] |
| k97-618294 | unclassified | transposase | IS481_family_transposase_[Bordetella_pertussis] |
| k97-1191397 | unclassified | transposase | IS110_family_transposase_[Paludisphaera_borealis] |
| k97-194759 | unclassified | recombinase | multifunctional_ATP-dependent_DNA_helicase /dsDNA/ssDNA_exonuclease/ ssDNA_endonuclease_recombinase_subunit [Pseudomonas_putida_KT2440] |
| k97-102563 | unclassified | transposase | transposase_[Cupriavidus_sp._HMR-1] |
| k97-933827 | unclassified | transposase | IS1380_family_transposase_[Streptococcus_pneumoniae] |
| k97-1277440 | unclassified | transposase | IS630_family_transposase_[Paenibacillus_sp._FSL_H7-0357] |
| k97-907392 | unclassified | transposase | MULTISPECIES:_Tn3_family_transposase_[Proteobacteria] |
| k97-1112807 | unclassified | transposase | transposase_[Geobacillus_sp._GHH01] |
| k97-874979 | unclassified | transposase | IS110_family_transposase_[Bradyrhizobium_sp.] |
| k97-1312805 | unclassified | transposase | Tn3_family_transposase_[Roseibium_sp._TrichSKD4] |
| k97-1774992 | unclassified | recombinase | tyrosine_recombinase_XerC_[Desmospora_sp._8437] |
| k97-1162988 | unclassified | transposase | transposase_[Rhizobium_etli] |
| k97-781296 | unclassified | transposase | transposase_[Cupriavidus_sp._HMR-1] |
| k97-519105 | unclassified | transposase | MULTISPECIES:_transposase_[Halomonas] |
| k97-791530 | unclassified | integrase | integrase_[Desulfatirhabdium_butyrativorans] |
| k97-869704 | unclassified | transposase | IS481_family_transposase_[Methylobacterium_sp._174MFSha1.1] |
| k97-1024290 | unclassified | recombinase | DNA_recombinase_[Rhodococcus_rhodochrous] |
| k97-213797 | unclassified | recombinase | recombinase_RecX_[Lutibacter_agarilyticus] |
| k97-367289 | unclassified | transposase | IS3_family_transposase_[Lactobacillus_parabuchneri] |
| k97-875176 | unclassified | recombinase | multifunctional_ATP-dependent_DNA_helicase/ dsDNA/ssDNA_exonuclease/ ssDNA_endonuclease_recombinase_subunit [Pseudomonas_putida_KT2440] |
| k97-875283 | unclassified | transposase | IS3_family_transposase_[Corynebacterium_aurimucosum] |
| k97-622707 | unclassified | transposase | IS1380_family_transposase_[Streptococcus_pneumoniae] |
| k97-1263764 | unclassified | transposase | MULTISPECIES:_IS3_family_transposase_[Streptomyces] |
| k97-594255 | unclassified | recombinase | recombinase_RmuC,_partial_[Listeria_monocytogenes] |
| k97-1115572 | unclassified | transposase | IS110_family_transposase_[Thermoanaerobacter_kivui] |
| k97-159535 | unclassified | recombinase | MULTISPECIES:_recombinase_A [Pseudomonas_stutzeri_subgroup] |
| k97-182368 | unclassified | recombinase | recombinase_A_[Fusobacterium_nucleatum_subsp._nucleatum_ATCC_25586] |
| k97-475017 | unclassified | transposase | ISL3_family_transposase_[Candidatus_Solibacter_usitatus] |
| k97-681976 | unclassified | transposase | MULTISPECIES:_transposase_[Rhodococcus] |
| k97-1051236 | unclassified | transposase | IS630_family_transposase_[Pseudonocardia_sp._EC080619-01] |
| k97-1298208 | unclassified | transposase | transposase_[Coprobacillus_sp._8_1_38FAA] |
| k97-870911 | unclassified | transposase | transposase_[Ruminococcus_flavefaciens] |
| k97-440867 | unclassified | transposase | IS66_family_transposase_[Leptolyngbya_sp._KIOST-1] |
| k97-145097 | unclassified | recombinase | recombinase_RarA,_partial_[Microbacterium_esteraromaticum] |
| k97-56527 | unclassified | integrase | integrase_[Rhizobium_arenae] |

**Table S2** Physical and chemical properties of soil and manure

|  | **pH** | **SOM** | **TN** | **TP** | **TK** | **AN** | **AP** | **AK** | **DOC** | **CEC** | **Particle Size Composition（%）** | | |
| --- | --- | --- | --- | --- | --- | --- | --- | --- | --- | --- | --- | --- | --- |
|  |  | **g/kg** | **g/kg** | **g/kg** | **g/kg** | **mg/kg** | **mg/kg** | **mg/kg** | **mg/kg** | **cmol/kg** | **2-0.05mm** | **0.05-0.002mm** | **<0.002mm** |
| **Ultisol** | 5.4±0.04 | 4.7±0.13 | 0.65±0.06 | 0.2±0.02 | 6.8±3.0 | 141.5±2.6 | 1.4±0.47 | 87.5±14.8 | 84.4±5.08 | 13.6±1.0 | 21.8±0.18 | 33.8±0.3 | 44.4±0.1 |
| **Mollisol** | 6.7±0.07 | 21.8±0.45 | 1.5±0.02 | 0.51±0.11 | 17.3±3.5 | 211.3±2.6 | 40.4±0.65 | 280±0.0 | 161.7±6.4 | 25±0.86 | 24.2±1.3 | 42.1±1.3 | 33.7±0.05 |

SOM, soil organic matter; DOC, dissolved organic carbon; CEC, cation exchange capacity

**Table S3** Contig assembly statistics

|  | **Contigs** | **Contigs bases(bp)** | **N50(bp)** | **N90(bp)** | **Max(bp)** | **Min(bp)** |
| --- | --- | --- | --- | --- | --- | --- |
| Mo-H1 | 1627740 | 1020163062 | 630 | 348 | 111217 | 300 |
| Mo-H2 | 1179471 | 761368385 | 664 | 353 | 99395 | 300 |
| Mo-H3 | 1481391 | 942762773 | 645 | 352 | 112157 | 300 |
| Mo-L1 | 1532676 | 963871144 | 625 | 348 | 154151 | 300 |
| Ul-H1 | 700100 | 563114797 | 927 | 370 | 150862 | 300 |
| Ul-H2 | 855879 | 679722812 | 906 | 368 | 185025 | 300 |
| Ul-H3 | 594690 | 517439265 | 1084 | 382 | 181891 | 300 |
| Ul-H4 | 607030 | 520875960 | 1060 | 380 | 186576 | 300 |
| Ul-L1 | 936774 | 741241721 | 907 | 367 | 212773 | 300 |
| Ul-L2 | 731039 | 611788006 | 1021 | 372 | 269760 | 300 |

**Table S4** Open reading frames assembly statistics

|  | **ORFs** | **Total Length(bp)** | **Average Length(bp)** | **Max(bp)** | **Min(bp)** |
| --- | --- | --- | --- | --- | --- |
| Mo-H1 | 2104330 | 930334506 | 442.1 | 15825 | 102 |
| Mo-H2 | 1547388 | 692982054 | 447.84 | 17301 | 102 |
| Mo-H3 | 1931798 | 859242843 | 444.79 | 17301 | 102 |
| Mo-L1 | 1950622 | 852076746 | 436.82 | 14550 | 102 |
| Ul-H1 | 1012929 | 499637604 | 493.26 | 20754 | 102 |
| Ul-H2 | 1229944 | 604014474 | 491.09 | 28239 | 102 |
| Ul-H3 | 894786 | 458873406 | 512.83 | 17319 | 102 |
| Ul-H4 | 905525 | 462630021 | 510.9 | 19455 | 102 |
| Ul-L1 | 1331999 | 642459177 | 482.33 | 24576 | 102 |
| Ul-L2 | 1073457 | 525562617 | 489.6 | 23739 | 102 |

**Figures**


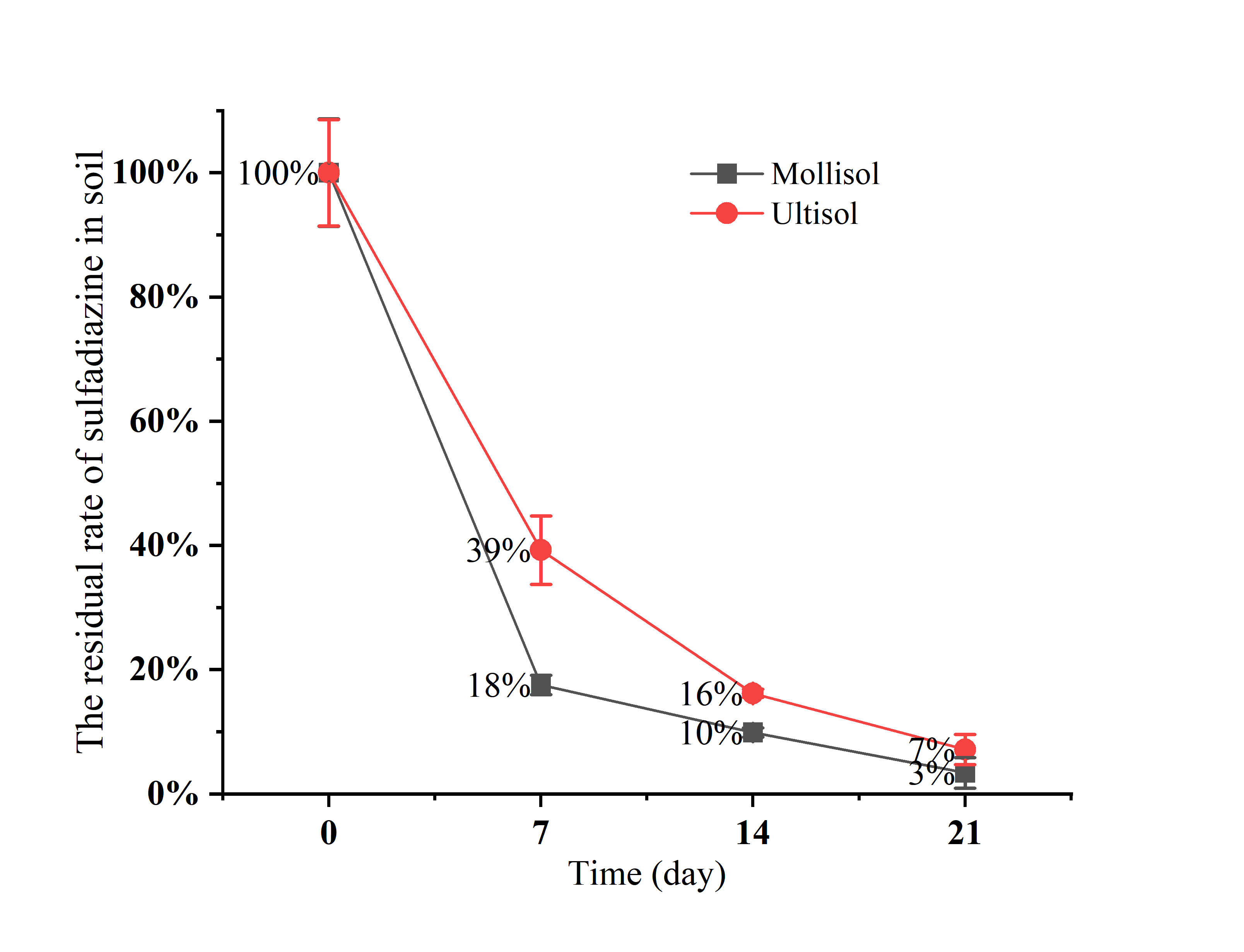


**Figure S1.** Degradation of sulfadiazine (SDZ) in the soil at different incubation periods.


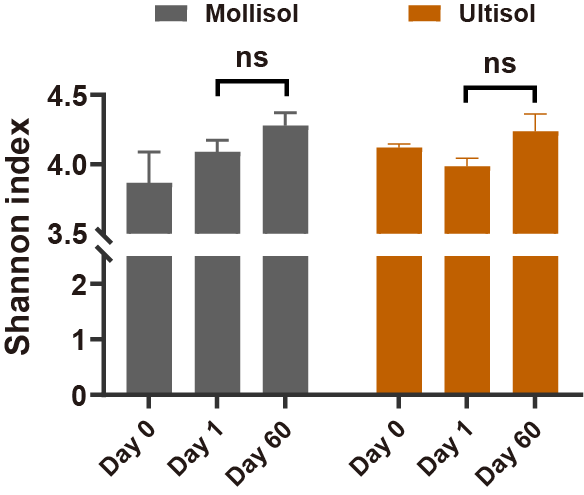


**Figure S2.** Shannon diversity index of the total microbial communities in antibiotic-treated Mollisol and Ultisol soils at Day 0, Day 1, and Day 60. No significant differences (ns) were observed between sampling days, indicating that the overall microbial diversity remained stable during incubation under antibiotic treatment.


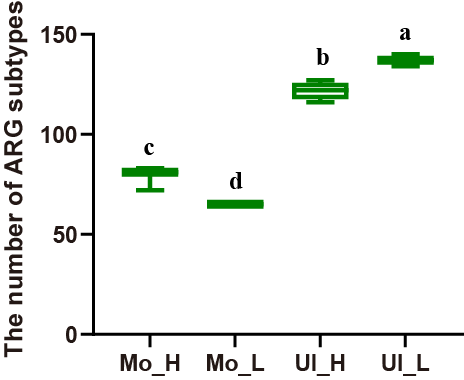


**Figure S3.** The number of antimicrobial resistance genes (ARG) subtypes across different soils. Mo_H: DNA of sulfadiazine-degrading bacteria; Ul_H: DNA of sulfadiazine-degrading bacteria; Mo_L: DNA of non-sulfadiazine-degrading bacteria; Ul_L: DNA of non-sulfadiazine-degrading bacteria. Different lowercase letters indicate significant differences among treatments (*p* < 0.05, Kruskal-Wallis test).


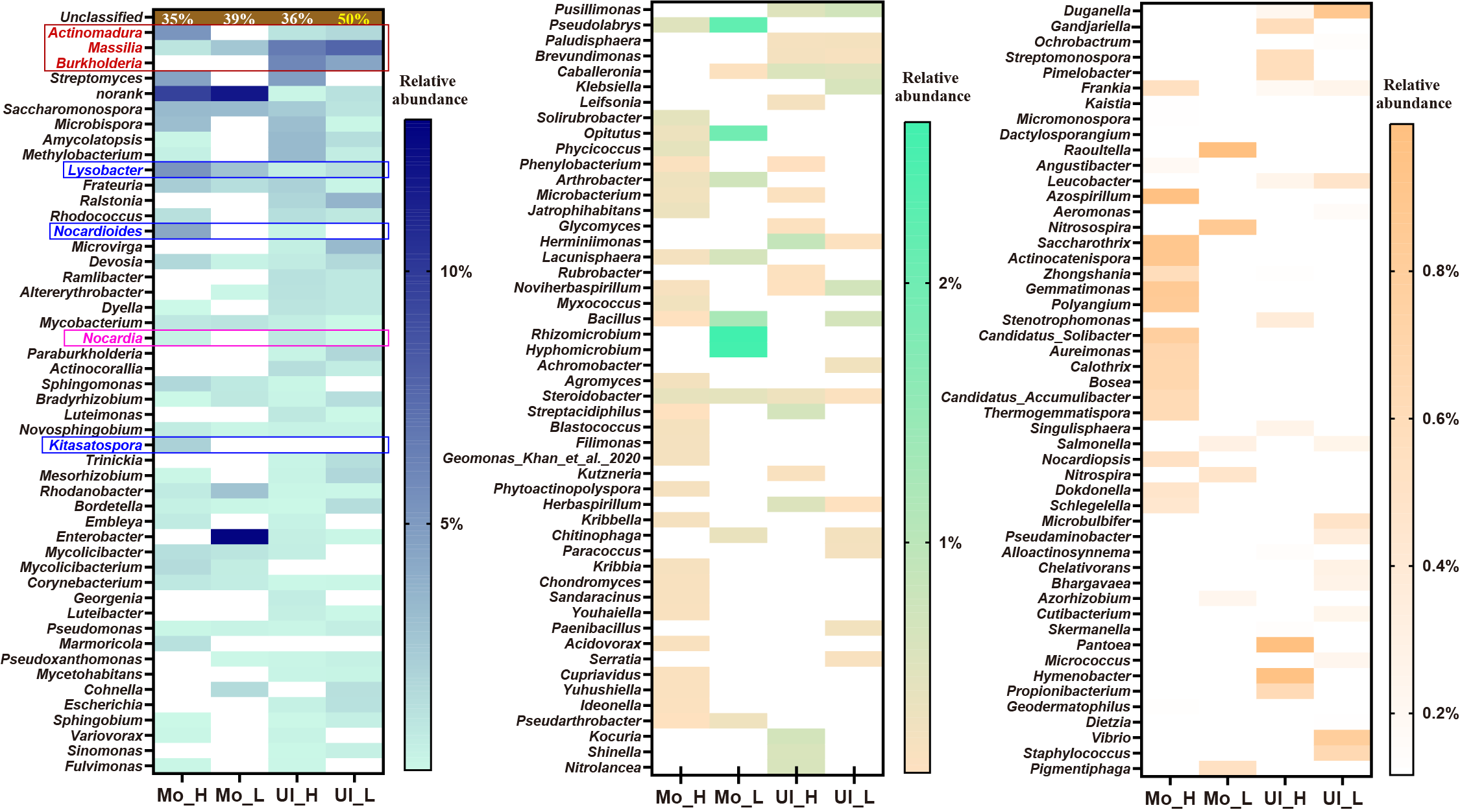


**Figure S4.** Relative abundance of ARG-hosting microorganisms at the genus level. Abbreviations of groups are the same as in Figure S2.

**
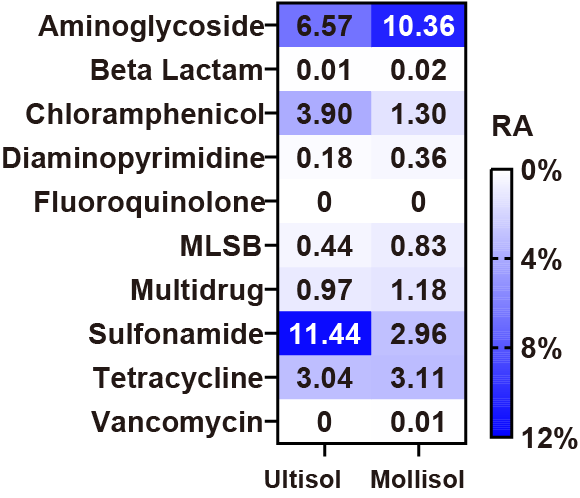
**

**Figure S5.** Relative abundance of ARGs of different antibiotic classes.

**
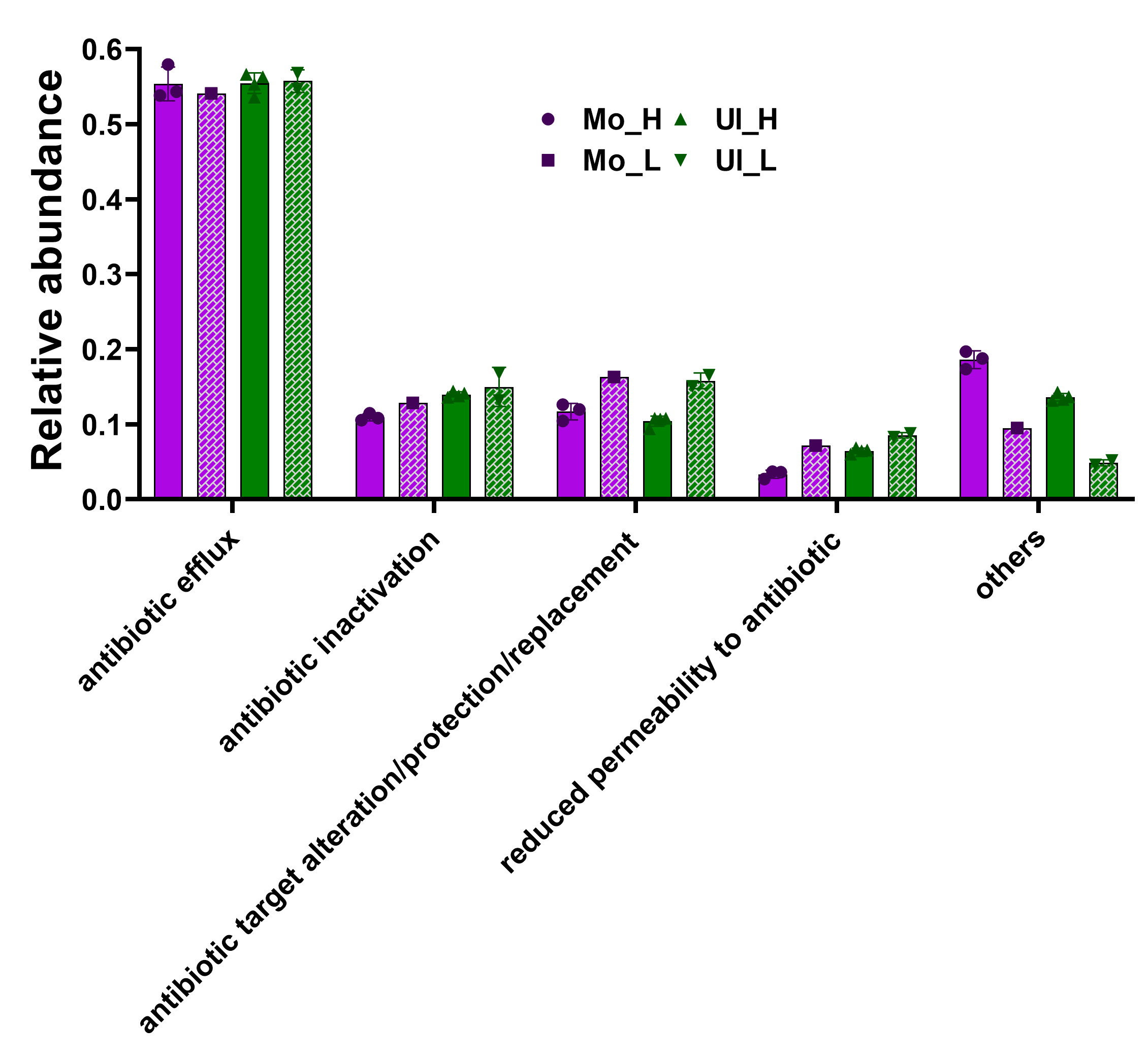
**

**Figure S6.** Relative abundance of ARGs of different mechanisms. Mo_H: DNA of sulfadiazine-degrading bacteria; Abbreviations of groups are the same as in Figure S2.
